# Supplementary material for: Co-Culture of P. gingivalis and F. nucleatum Synergistically Elevates IL-6 Expression via TLR4 Signaling in Oral Keratinocytes
Source: Int J Mol Sci. 2024 Mar 23;25(7):3611. doi: 10.3390/ijms25073611 (PMC11011619; doi:10.3390/ijms25073611)
Supplement: Supplementary file 1 [file ijms-25-03611-s001.zip › ijms-2909097-supplementary.pdf]

SUPPLEMENTARY FIGURES

Supplementary figure 1 (Figure S1): Schematic illustrating the differences between the co-culture and the co-infection conditions for *P. gingivalis* and *F. nucleatum*.

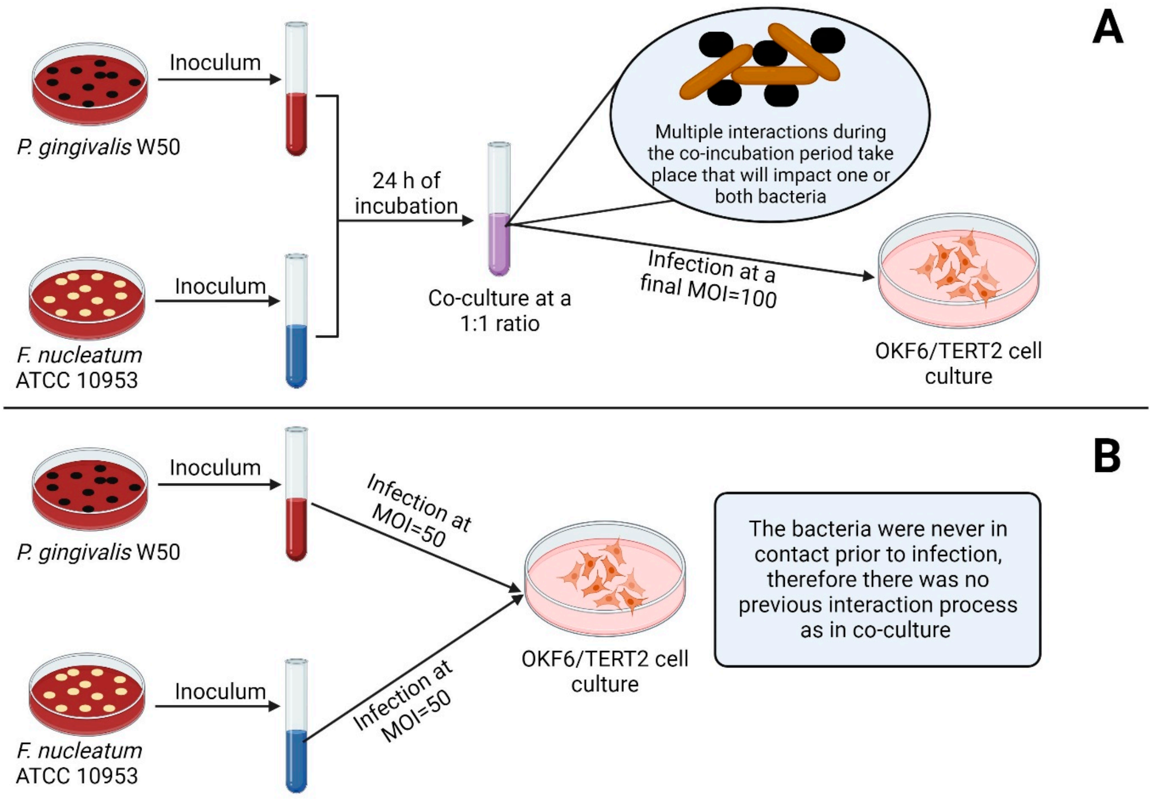

Figure S1. Schematic of the differences between the co-culture and the co-infection conditions for *P. gingivalis* and *F. nucleatum*. The schemes show the experimental design for the infection with the co-culture **(A)** and the co-infection **(B)** with separately grown *P. gingivalis* and *F. nucleatum*. All infections were done at a final virtual MOI = 100.

Supplementary figure 2 (Figure S2): Schematic illustrating the differences between the co-culture and the co-infection conditions for *P. gingivalis* and *F. nucleatum*.

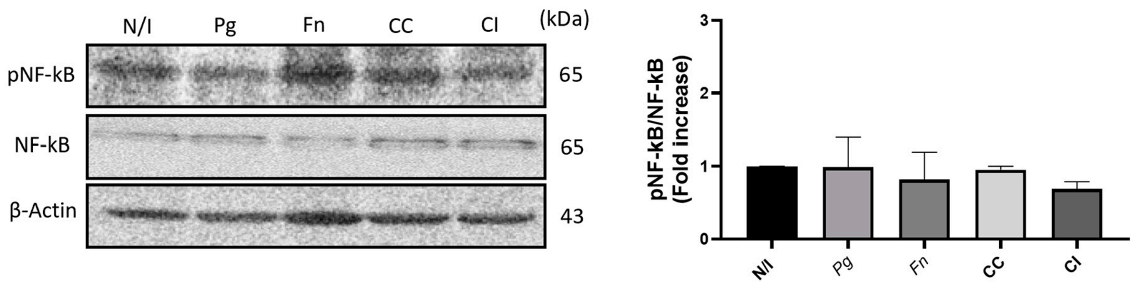

Figure S2. The co-culture of *P. gingivalis* and *F. nucleatum* does not affect the phosphorylation levels of NF-kB 24 h post infection.

Representative images of **(A)** phospho-NF-kB(S536) and total NF-kB by Western blot analysis on OKF6/TERT2 cell extracts 24 h post infection (left panel) and the respective quantifications by scanning densitometry (right panel) are shown. Data were analyzed by one-Way ANOVA, followed by a Dunnett post-test (n=3). **N/I** = non-infected control; ***Pg*** = *P. gingivalis*; ***Fn*** = *F. nucleatum*; **CC** = co-culture; **CI** = co-infection with both bacteria previously grown separately.

Supplementary figure 3 (Figure S3): Growth curves of *P. gingivalis* and *F. nucleatum* in supplemented BHI/Cys medium.

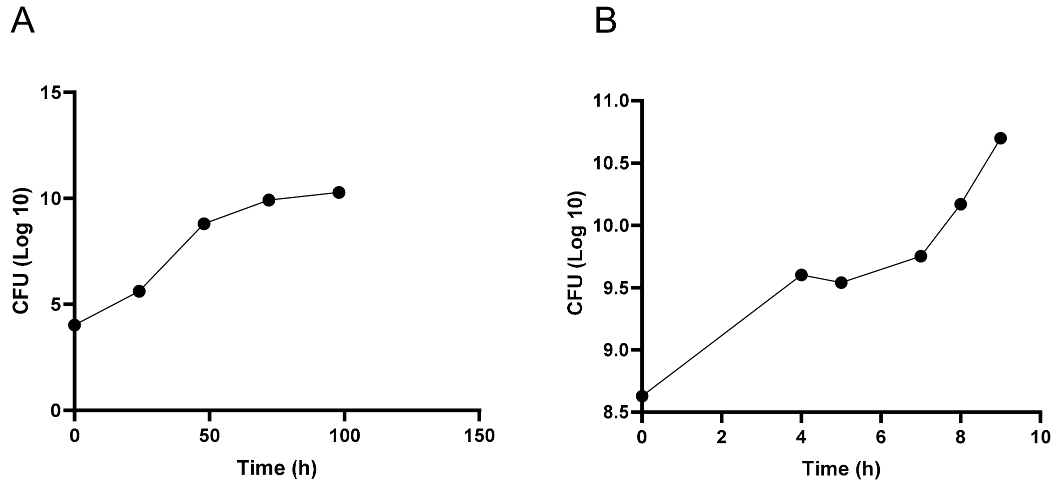

Figure S3: Growth curves of *P. gingivalis* and *F. nucleatum*. Growth curves of the **(A)** *Porphyromonas gingivalis* strain W50 and **(B)** *Fusobacterium nucleatum* ATCC 10953 are shown. Both bacteria were cultured separately in BHI/Cys broth supplemented with 1% hemin and 1% menadione and the OD_{600} was measured after 1 h of incubation at 37°C in anaerobiosis. Bacteria were seeded on blood agar plates previously supplemented with 1% hemin and 1% menadione when exponential phase was reached. Then, CFU/mL obtained for *F. nucleatum* and *P. gingivalis* were counted after incubating for 2 or 5 days, respectively (n=3).

Supplementary figure 4 (Figure S4): Effects of the co-culture of *P. gingivalis* and *F. nucleatum* on NF-kB levels in shTLR4 cells.

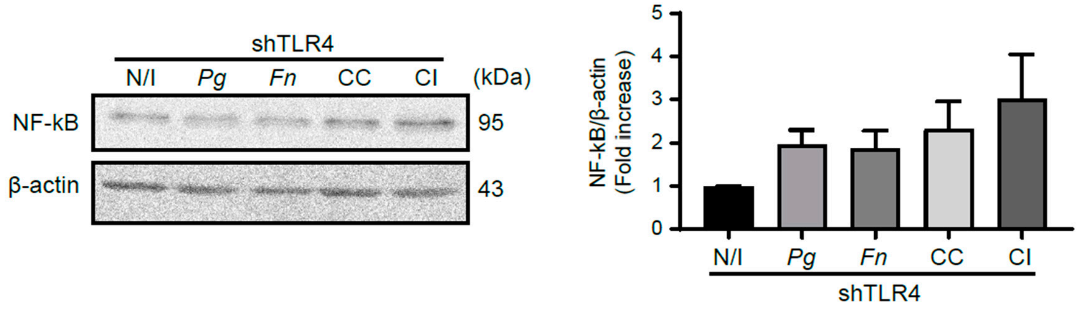

Figure S4. Effects of the co-culture of *P. gingivalis* and *F. nucleatum* on NF-kB levels in shTLR4 cells. Representative images of **(A)** NF-kB Western blot analysis of shTLR4 cells 24 h post-infections and (B) the respective quantification by scanning densitometry. Data were analyzed by one-Way ANOVA, followed by a Dunnet's post-test, (* $p<0.05$; n=3). **NI** = Non-infected control; ***Pg*** = *P. gingivalis*; ***Fn*** = *F. nucleatum*; **CC** = co-culture; **CI** = co-infection with bacteria grown individually.

Supplementary figure 5 (Figure S5): Expression of pro-inflammatory cytokines in shScrambled cells 24 h post infection.

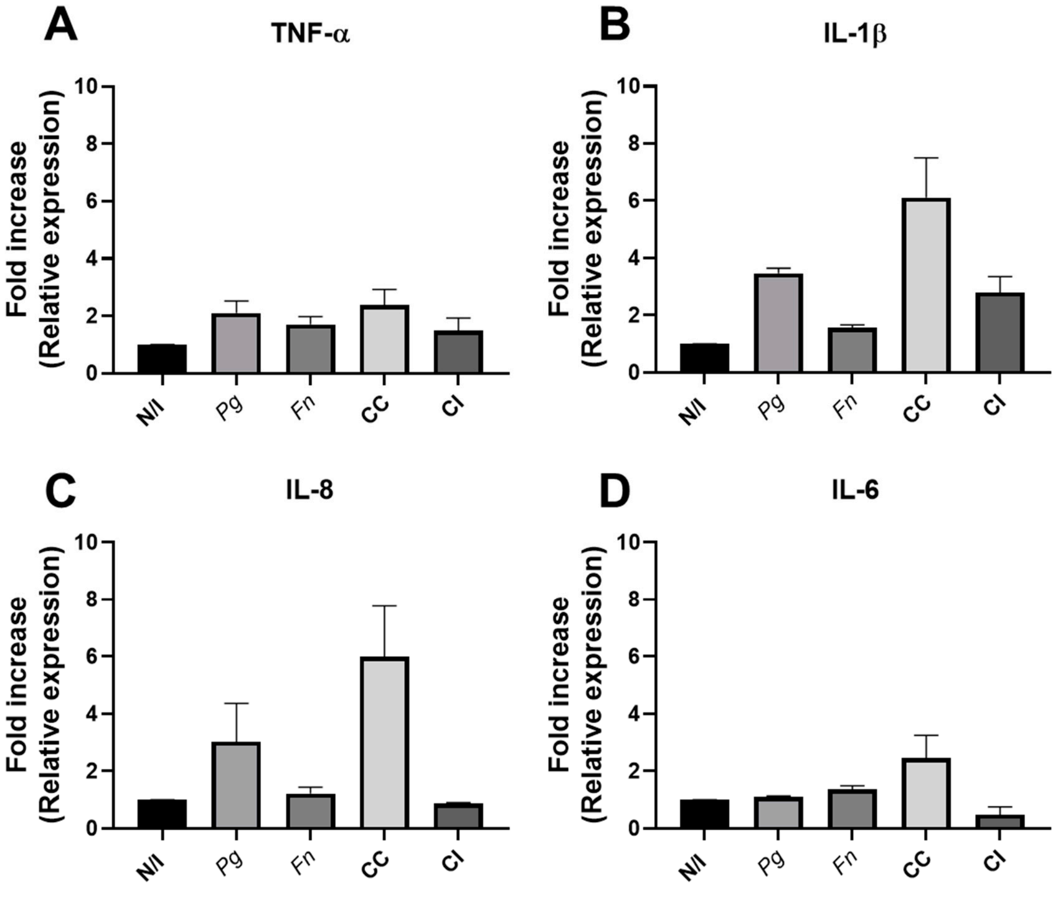

Figure S5. Expression of pro-inflammatory cytokines in shScrambled cells 24 h post infection. The fold increase in the relative expression (ΔΔCT) of pro-inflammatory cytokines TNF-α (A), IL-1β (B), IL-8 (C) and IL-6 (D) in shScramble cells, 24 h post infection, is shown. n=2. N/I = non-infected control; Pg = *P. gingivalis*; Fn = *F. nucleatum*; CC = co-culture; CI = co-infection with both bacteria previously grown separately.
